# Academic Collaboration Results in a Meaningful Change

**DOI:** 10.12669/pjms.41.13(PINS-NNOS).14602

**Published:** 2025-12

**Authors:** Asif Bashir

**Affiliations:** 1 Asif Bashir, MD, FAANS, FACS Diplomate, American Board of Neurological Surgery Professor & Chair, Neurosurgery Executive Director & Dean Punjab Institute of Neurosciences (PINS) Lahore General Hospital (LGH), Lahore, Pakistan

In a nation like Pakistan, where progress often faces the weight of limited resources, institutional silos, and systemic challenges, collaboration is not just helpful: it is essential. Every step forward in our healthcare and academic systems has come not from isolated effort, but from people and institutions willing to work together for a common purpose. It is through this spirit of collaboration that meaningful change becomes possible.

That belief lies at the heart of this publication, the Punjab Institute of Neurosciences National Neuro-Oncology Supplement (PINS-NNOS). It is the product of trust, shared vision, and an ongoing partnership between PINS and the Pakistan Journal of Medical Sciences (PJMS), now joined by the Pakistan Society of Neuro-Oncology (PASNO) as well. Together, we have once again created a platform for the best of our nation’s neurosciences to be seen, shared, and celebrated.

When we first launched the PINS–PJMS Supplement 2024, our goal was simple but ambitious: to showcase the academic and clinical growth taking place at PINS, and to affirm that institutions in Pakistan can also produce research of international standard when given direction and opportunity.^1^ The response exceeded every expectation. The supplement was widely read, cited, and praised for its academic rigor and institutional coherence. It became a benchmark for how public-sector medical institutions could contribute meaningfully to the body of scientific literature.

Building on that momentum, the year since has been one of expansion, both in scope and spirit. Within PINS, we achieved a long-envisioned goal with the establishment of a second Neurology Unit: Vascular Stroke, completing our foundation as a comprehensive neurosciences center. This milestone has strengthened our ability to deliver multidisciplinary care and to train the next generation of neurologists and neurosurgeons within a unified institutional framework. We also inaugurated the Almas Bashir Research and Editorial Office this year, a modern and dedicated hub for our academic and research activities.^2^ Our research output has continued to grow, supported by a culture that values inquiry and innovation.

Our ongoing collaborative research projects are central to that evolution. We are taking part in the Randomised Evaluation of Surgical teChniques for patients Undergoing Emergency Cranial Decompression (RESCUE CD) with Cambridge University, linking our clinicians and researchers with an international network dedicated to improving trauma outcomes. The prospective Pakistan Brain Tumor Epidemiology Study (pPBTES), in partnership with the Aga Khan University (AKU), is another pioneering effort. Each of these collaborations tells a story of what can be achieved when institutions choose partnership over isolation.

This year also marked another first: the inaugural PINSCON 2025, held from October 18–21, 2025. This congress brought together experts, trainees, and allied professionals from across Pakistan and abroad, reflecting the same collaborative ethos that defines PINS itself. It was a space for shared learning, bridging disciplines, and strengthening the professional community that sustains neurosurgical and neuro-oncological care in our country. World-renowned faculty such as Fady T. Charbel, Head of the Department of Neurosurgery at the University of Illinois at Chicago, Charles L. Branch, Chair of Neurosurgery at the Wake Forest University School of Medicine and President of the North American Spine Society (NASS), and David Hasan, Professor of Neurosurgery and Endovascular Surgery at Duke University, were present to give lectures and experience our country for the first time, along with medical professionals from every corner of Pakistan.

Fittingly, this year’s supplement focuses on neuro-oncology, a field that demands precisely that kind of collective effort. Developed in close collaboration with PASNO, the PINS National Neuro-Oncology Supplement 2025 includes contributions from across Pakistan’s public and private institutions. The 86 submissions we received spanned a remarkable range of disciplines: neurosurgery, neurology, radiology, pathology, oncology, anesthesia, critical care, rehabilitation, palliative care, and nursing. After rigorous peer review, thirty-six high-quality manuscripts were accepted for publication. Together, they form one of the most comprehensive academic supplements on neuro-oncology ever produced from a low- and middle-income country.

I wish to express my deep gratitude to everyone who made this possible: to our peer reviewers, whose expertise and diligence ensured scientific integrity; to Mr. Shaukat Ali Jawaid, Chief Editor of Pakistan Journal of Medical Sciences, for his unwavering support and editorial guidance; and to Prof. Syed Ather Enam, President of PASNO, and its Executive Council for their commitment to advancing neuro-oncology education and research nationwide. Lastly, to Dr. Haseeb Mehmood Qadri, our outstanding neurosurgery resident, whose unwavering dedication and indispensable contributions have been instrumental in making this supplement—and the continued progress of PINS—possible. I deeply appreciate and wholeheartedly acknowledge his exemplary commitment.

At its core, this publication is about more than academic output. It is a reflection of who we are and what we aspire to be, a community of professionals bound by purpose, striving to bring world-class care and research to our people, despite the odds. PINS stands today as a living example of what institutional collaboration can achieve when driven by vision, persistence, and faith.

May this spirit continue to guide us as we build the future of neurosciences in Pakistan, one partnership, one publication, one patient at a time.

## References

[1] Pakistan Journal of Medical Sciences - Punjab Institute of Neurosciences (PINS) Supplement 2024.

[2] Almas Bashir Research and Editorial Office (REO) Inaugurated at PINS Pulse Pakistan Int.

